# Risk factors of paternal postnatal depression in Pakistan: Findings from an urban sample

**DOI:** 10.1111/nhs.12954

**Published:** 2022-06-03

**Authors:** Maria Atif, Mark Halaki, Chin Moi Chow, Camille Raynes‐Greenow

**Affiliations:** ^1^ School of Public Health, Faculty of Medicine and Health The University of Sydney Sydney New South Wales Australia; ^2^ Discipline of Exercise and Sport Science, Faculty of Medicine and Health The University of Sydney Sydney New South Wales Australia

**Keywords:** men, mental health, Pakistan, perinatal, postnatal depression

## Abstract

Paternal postnatal depression is an emerging public health concern, with negative outcomes for men, their partners, and the newborn. There is a dearth of data on paternal postnatal depression in lower‐middle‐income countries like Pakistan. This study aimed to identify risk factors of postnatal depression in Pakistani men. Men who consented to this cross‐sectional study completed a questionnaire that included sociodemographic information and Urdu translated versions of the Edinburgh Postnatal Depression Scale (EPDS) and the Pittsburgh Sleep Quality Index, 10–12 weeks postpartum. Descriptive analyses for the sociodemographic variables were calculated. Univariate analyses were conducted to calculate the relative risk and 95% confidence interval of the independent variables with an EPDS score of >10. Multivariate binary logistic regression models were performed for risk factors of paternal postnatal depression. Fifty‐one questionnaires were analyzed and 23.5% of the participants scored more than 10 on the EPDS. Spouse's EPDS score > 12, and own sleep disturbance were risk factors of paternal postnatal depression in Pakistani men. There is an imminent need to incorporate fathers in the existing and future perinatal mental health programs in Pakistan.

**Key points**

- Paternal postnatal depression is underresearched, especially in lower‐middle‐income countries like Pakistan.
- This study, for the first time, identified the risk factors of paternal postnatal depression in Pakistani men and found that 23.5% of the participants screened positive for depression on the Edinburgh Postnatal Depression Scale.
- We found positive associations between paternal and maternal depression and between paternal postnatal sleep disturbance and paternal postnatal depression in Pakistani men.

## 1 INTRODUCTION

Depression is one of the most common mental health morbidities around the world (Lim et al., 2018; Sinyor et al., 2016), with negative consequences for the individual and the community (Top et al., 2016). According to a recent meta‐analysis that included one million participants from 30 countries, the aggregate point prevalence of depression was 12.9%, the 1‐year prevalence was 7.2%, and lifetime prevalence was 10.8% from 1994 to 2014 (Lim et al., 2018). The postnatal period (also referred to as the postpartum period), extends until 1 year after birth (Garcia & Yim, 2017). This period involves considerable physiological and psychosocial changes and is recognized as a period of vulnerability for new parents to be affected by depression (Nillni et al., 2021; Vismara et al., 2016).

Issues related to birth have been historically associated with women and there is an abundance of data on maternal postnatal depression; however, recently paternal postnatal depression has emerged as a public health concern and a major research gap (Garthus‐Niegel et al., 2020; Philpott & Corcoran, 2018). The available literature suggests that postnatal depression can also affect men, and the prevalence of paternal depression may range from 9.76% during the antenatal period to 8.75% during the first year following birth (Rao et al., 2020). The detection of paternal postnatal depression remains a challenge because of the stigma associated with issues about mental health in men (Shaheen et al., 2019). This stigma may cause men to suppress their feelings and make them less likely to acknowledge their depressive symptoms (Shaheen et al., 2019), which might further contribute to their depression. Although recognized as a major depressive disorder like maternal postnatal depression, paternal depression has subtle differences in its manifestation as compared to maternal depression. In contrast to the most common symptoms of maternal depression, including feelings of loneliness, insecurity, anxiety, loss of control, guilt, diminished concentration, lack of positive emotions, loss of interest in hobbies or goals, and contemplation of harming themselves and their infants, the paternal postnatal depression symptoms may be characterized by anger attacks or aggression, irritability, substance abuse, and risk‐taking behaviors (Affleck et al., 2018; Martin et al., 2013), making it a challenge to detect paternal depression.

Paternal postnatal depression is an emerging public health concern because of its potential to affect not only the man but also his intimate partner and the newborn (Fletcher et al., 2017; Stein et al., 2014). Paternal postnatal depression is considered one of the most significant risk factors for long‐term adverse impacts on the emotional, behavioral, cognitive, and physical health of the child (Stein et al., 2014). The exact etiology of paternal postnatal depression remains debatable; however, several risk factors have been identified in the global literature (Cui et al., 2021; Da Costa et al., 2019) Maternal depression is recognized as one of the strongest risk factors of depression in fathers during the postnatal period (Zhang et al., 2016). The relationship between maternal and paternal depression can be bidirectional, with paternal depression also being a risk factor for maternal depression (Thiel et al., 2020). Besides maternal depression, numerous studies have also identified economic factors, such as low income and unemployment as some of the risk factors of paternal postnatal depression (Carlberg et al., 2018; Da Costa et al., 2019). Sleep disturbance, marital conflict, low income, unplanned pregnancy, preference for a male child, antenatal depression, and a history of previous psychiatric illness have also been implied as possible risk factors for paternal postnatal depression (Chhabra et al., 2020; Kalogeropoulos et al., 2021).

With an estimated population of 220 million, Pakistan belongs to the lower‐middle‐income group of countries. Pakistan has a high prevalence of depressive disorders, ranging between 22% and 60% (Nisar et al., 2019). Mental health issues are highly stigmatized in Pakistani culture (Waqas et al., 2014). Pakistani men suffering from mental illnesses are especially challenged not only by their illness but also by the associated stigma, and symptoms of depression remain inconsistent with the prevalent concepts of masculinity in Pakistani society (Affleck et al., 2018; Waqas et al., 2014). There is a dearth of literature on paternal depression in Pakistan. A recent study estimated the prevalence of paternal postnatal depression to be 28.3% (Noorullah et al., 2020); however, to the best of our knowledge no data are available on possible risk factors of paternal postnatal depression in Pakistan. This study, therefore, aims to analyze the risk factors of paternal postnatal depression in Pakistan.

## 2 METHODS

### 2.1 Study design, setting, and sample

This observational study was conducted in the Fatima Bai Hospital Karachi, Pakistan between December 2018 and July 2019. Previous studies from Pakistan have reported that pregnancy is usually considered a feminine matter in Pakistan; therefore, men are excluded from their wives' reproductive health issues, including antenatal care visits (Noh et al., 2019). Considering the sociocultural construct of Pakistani society, and the unavailability of other studies on paternal postnatal depression from Pakistan to guide our data collection methodology, we decided to recruit study participants through their wives, waiting to attend antenatal care. Participants' information statements and consent forms were distributed to 73 women who agreed to pass them to their husbands. Men who consented to the study returned the consent forms either directly to the researcher (at the participating hospital) or through their spouses. By consenting to the study, the participants acknowledged that they would be contacted via telephone, approximately 10–12 weeks after the birth. For this purpose, the data collector noted the expected date of delivery of each participant's spouses and recorded two telephone numbers. Based on the expected date of delivery of participants' spouses, the data collector informed participants of a tentative period, during which the postnatal data would be collected. Between 10–12 weeks after the birth of their child, participants completed a self‐report questionnaire that collected sociodemographic data, the Edinburgh Postnatal Depression Scale (EPDS), and the Pittsburgh Sleep Quality Index (PSQI). To reduce the risk of information bias, the participants were ensured of the confidentiality of their information through anonymization, and to reduce the risk of recall bias the data were collected prospectively during the postnatal period.

### 2.2 Data collection

The self‐report questionnaire consisted of sociodemographic items, including the financial situation of the family, interpersonal relationships, adverse life events (death/illness/separation from a loved one or loss of job), history of smoking and drug use, the recent birth experience, the well‐being of the newborn, and knowledge of postnatal depression. EPDS was used to screen men for postnatal depression. The EPDS is a 10‐item self‐reporting questionnaire, which is predominantly used for screening postnatal depression in the female population. However, EPDS has also been validated to screen depression in men (Edmondson et al., 2010). For this study, a score of >10 was used as a cutoff value for screening positive for depression and therefore, considered depressed and referred to as “postnatal depression.” We used an Urdu translated version of the EPDS, which was validated for the female population (Husain et al., 2014). The reliability of this tool for screening paternal depression was determined by piloting it on 10 men, which yielded a Cronbach's alpha of 0.78. The PSQI translated into Urdu and validated for Pakistan was used to assess sleep quality and disturbance (Hashmi et al., 2014). The PSQI consists of 19 self‐rated questions, related to participants' usual sleep habits during the past month. A global score of 5 or more indicates poor sleep quality (the higher the score, the worse the sleep quality). Permission was sought from the authors to use the Urdu translated versions of the questionnaires for this study.

### 2.3 Data analysis

Descriptive analyses for the sociodemographic variables were calculated. Results were expressed as mean and standard deviation for normally distributed data and median and interquartile range (IQR) for non‐normally distributed data. The normality of EPDS and PSQI scores was assessed by the Kolmogorov‐Smirnov test (EPDS sig. 0.01, PSQI sig. <0.00). Variables were assessed for multicollinearity, and the data met the assumption of collinearity with variance inflation factor <5 for all independent variables. Prevalence estimates of depression, based on EPDS scores were calculated. The associations were reported as relative risks (RRs) with 95% confidence intervals (CIs) and the level of significance was set at *p* < 0.05. Variables, including age, employment status, self‐reported financial hardship, number of children, own and spouse's sleep disturbance, spouse screened positive for perinatal depression, and satisfaction with the marital relationship were considered for multivariate binary logistic regression analysis in a single predictor model (Enter mode) to assess their individual predictive ability while controlling for the effects of other variables in the model. Significance was set at *p* value <0.05 and associations were reported as adjusted odds ratios with 95% CIs. Statistical analyses were performed using IBM SPSS statistics for windows, Version 28.0. (IBM Corp., Armonk, NY, USA).

The ethics review board of Fatima Bai Hospital provided ethical approval (No: RD‐056‐APP‐RRB‐0034‐2018).

## 3 RESULTS

Out of 73 participants, approached via their spouses, 64 returned the consent forms to the data collector (87%). However, only 51 of these participants could be reached by telephone 10–12 weeks postpartum for data collection. Therefore, 51 questionnaires were included in the data analysis.

During the postnatal period, 23.5% of the participants scored more than 10 on the EPDS (median = 7, IQR = 7), whereas 33% scored 5 or more on the PSQI (median = 4, IQR = 5). The mean ± standard deviation age of the participants was 31 ± 5.2 years. More than half of the participants received formal education (55%) and 92% reported being employed. Approximately half of the participants (54.9%) reported a monthly family income of less than Pakistani rupee (PKR) 50000 (equivalent to ≈ USD 285) and 33% reported facing current financial difficulties. A majority of the participants lived in extended families (84%) and 67% reported having more than one child. Almost half of the participants (45%) reported the recent pregnancy to be unplanned, and only 8 participants (16%) had any knowledge of postnatal depression (Table 1).

**TABLE 1:** Demographic characteristics of the participants in the analyzed sample in Karachi, Pakistan

Demographic variables	Characteristics of the participants N = 51 (%)
Age (years)	
<30	22 (43.2)
≥30	29 (56.8)
Employed	
Yes	47 (92.2)
No	4 (7.8)
Received formal education	
Yes	28 (54.9)
No	23 (45.1)
Monthly family income	
<20 000	5 (9.8)
20 000–<30 000	17 (33.3)
30 000–<50 000	6 (11.8)
50 000–<75 000	7 (13.7)
75 000–<100 000	4 (7.8)
≥100 000	12 (23.5)
Living in nuclear family	
Yes	8 (15.7)
No	43 (84.3)
Financial hardship	
Yes	19 (33.3)
No	32 (66.7)
Children	
Have only one child (newborn)	17 (37.2)
Have multiple children (including newborn)	34 (66.7)
Unplanned pregnancy	
Yes	23 (45.1)
No	28 (54.9)
Preference for baby's sex	
Male	22 (43.1)
Female	8 (15.7)
None	21 (41.2)
Knowledge about postnatal depression	
Yes	8 (15.7)
No	43 (84.3)

In the univariate analysis, spouse's EPDS >12 (RR = 6.59; 95% CI = 1.60, 27.08), their own sleep disturbance (RR = 6.00; 95% CI = 1.86, 19.32), spouse's sleep disturbance (RR = 3.65; 95% CI = 1.11, 11.93), aged <30 years (RR = 2.70; 95% CI = 1.58, 4.62), satisfaction with the marital relationship (RR = 2.59; 95% CI = 1.02, 6.60), and financial hardship (RR = 5.05; 95% CI = 1.55, 16.39) were all risk factors of postnatal depression (Table 2). Moreover, employed men were less likely to be depressed than their counterparts were (odds ratio [OR] = 0.88; 95% CI = 0.00, 3.81). However, only spouse's EPDS score > 12 (OR = 13.04; 95% CI = 1.82, 28.37), and own sleep disturbance (OR = 9.40; 95% CI = 1.09, 21.62) remained significant risk factors in the model for paternal postnatal depression in the final multivariate analysis (Table 2).

**TABLE 2:** Risk factors of paternal postnatal depression in Karachi, Pakistan

Characteristics of the participants (N = 51)	EPDS score ≤ 10 (N = 39)	EPDS >10 (N = 12)	Univariate analysis RR (95% CI)	Multivariate analysis Exp. B (95% CI)
Age < 30 years				
Yes	12 (54.5%)	10 (45.5%)	2.70 (1.58, 4.62)*	3.84 (0.17, 8.01)
No	27 (93.1%)	2 (6.9%)	reference	reference
Employed				
Yes	38 (80.9%)	9 (19.1%)	0.25 (0.11, 0.57)*	0.88 (0.00, 3.81)
No	1 (25.0%)	3 (75.0%)	reference	reference
Financial hardship				
Yes	10 (52.6%)	9 (47.4%)	5.05(1.55, 16.39)*	7.71 (0.31, 19.85)
No	29 (90.6%)	3 (9.4%)	reference	reference
Children				
Have only one child (newborn)	14 (82.4%)	3 (17.6%)	1.50 (0.46, 4.83)	8.14 (0.32,21.87)
Have multiple children (including newborn)	25 (73.5%)	9 (26.5%)	reference	reference
Sleep disturbance				
Yes	8 (47.1%)	9 (52.9%)	6.00 (1.86, 19.32)*	9.40 (1.09, 21.62)*
No	31 (91.2%)	3 (8.8%)	reference	reference
Spouse's sleep disturbance				
Yes	14 (60.9%)	9 (39.1%)	3.65 (1.11, 11.93)*	5.58 (0.70, 14.13)
No	25 (89.3%)	3 (10.7%)	reference	reference
Spouse screened positive for depression				
Yes	12 (54.5%)	10 (45.5%)	6.59 (1.60, 27.08)*	13.04 (1.82, 28.37)*
No	27 (93.1%)	2 (6.9%)	reference	reference
Satisfaction with marital relationship				
Yes	33 (82.5%)	7 (17.5%)	2.59 (1.02, 6.60)	0.23 (0.03, 1.73)
No	6 (54.5%)	5 (45.5%)	reference	reference

Abbreviations: CI, confidence interval; EPDS, Edinburgh Postnatal Depression Scale; RR, relative risk.

* *p* < 0.05.

## 4 DISCUSSION

In this study, almost a quarter of the participants screened positive for depression on EPDS (score more than 10). The risk factors of paternal postnatal depression were maternal depression (having a spouse with EPDS score > 12) and paternal sleep disturbances during the postnatal period. Having a satisfactory relationship with a spouse and facing financial hardship were predictors of paternal postnatal depression in the univariate analysis but did not contribute to the final model.

The prevalence estimate for paternal postnatal depression in this study is congruent with that reported by Noorullah et al. in their recent cross‐sectional study in Karachi, Pakistan (Noorullah et al., 2020), but higher compared to those reported by earlier meta‐analyses(Cameron et al., 2016; Rao et al., 2020), which primarily included studies conducted in developed countries. Similar discrepancies have been observed in studies reporting on prevalence estimates of maternal depression suggesting that parental postnatal depression is more prevalent in resource‐constrained, low‐income countries (Hahn‐Holbrook et al., 2018; Wang et al., 2021). Adverse economic circumstances have been previously implicated as one of the possible risk factors of paternal postnatal depression (Carlberg et al., 2018; Da Costa et al., 2019), and although we did not find a significant association between financial hardship and postnatal depression in Pakistani men, the findings of this study should be interpreted with caution due to small sample size.

This study found that maternal postnatal depression was a risk factor of paternal postnatal depression in Pakistan, consistent with studies that reported similar positive associations(Nishimura et al., 2015; Zhang et al., 2016); however, a temporal relationship between the two has not been established (Thiel et al., 2020). Shared environmental and interpersonal stressors have been implied to affect both partners, which may ultimately contribute to the development of depressive symptoms (Thiel et al., 2020). Because the postnatal period requires several adjustments in daily life, men may rely on their partner for emotional support after the birth of their child and this support may become a challenge if the other partner is also experiencing postnatal depressive symptoms. Men who feel less supported by their partners, perceive more change in their partners post birth, or experience marital dissatisfaction during the postnatal period, may find it more difficult to adjust to their new role as a father. In the patriarchal society of Pakistan, there is a clear demarcation between gender roles, based on traditional and social values (Islam & Asadullah, 2018). For instance, men in Pakistan are generally considered the breadwinners of the family and are not expected to be involved in the care of children, especially at night (Jeong et al., 2018). Men are often stereotyped as physically strong, assertive, and objective beings, responsible to protect and provide for their families, and any expression of vulnerability is not socially acceptable (Nisar et al., 2019). However, the finding of this study that men may also experience postnatal depression highlights a need to include fathers in the existing and future perinatal care and management programs.

A link between sleep disturbances and paternal postnatal depression has been well documented (Da Costa et al., 2015; Kalogeropoulos et al., 2021). This study also found sleep disturbance to be a risk factor of paternal postnatal depression in Pakistani men. Kalogeropoulos et al. investigated the association between sleep and postnatal depressive symptoms in 54 Canadian fathers (previously not diagnosed with depression) using subjective and objective sleep measures and found that self‐reported perceived sleep quality was significantly associated with paternal postnatal depression, with first‐time fathers more likely to suffer from depression due to sleep disturbances in the postnatal period (Kalogeropoulos et al., 2021). Despite not having a direct role in caring responsibilities of the newborn, 33% of the fathers in our study reported sleep disturbances during the postnatal period. This finding requires further research to examine factors that may affect paternal sleep during the postnatal period. Such knowledge may enable us to plan intervention programs to address sleep‐related perinatal mental health challenges.

We acknowledge several limitations of this study. First, the data collection method, although the most feasible and cost‐effective method for us, may have introduced bias since participants were recruited through their partners. Therefore, a more independent recruitment method is suggested for future research. Further, the small sample size limits the generalizability of the findings to other similar settings. Future research with a larger sample would improve generalizability. Data were collected cross‐sectionally during the postnatal period and are therefore subject to the usual limitations of cross‐sectional data, such as temporality. Moreover, the use of EPDS, a screening tool, may have overestimated the true prevalence of paternal depression, although our data are consistent with other estimates of paternal postnatal depression (Noorullah et al., 2020). Despite these limitations, this study was unique as it attempted to analyze the risk factors of paternal depressive symptoms, acknowledging the existence of this mental health issue in Pakistani men and adding to our limited knowledge of this problem in Pakistan. Findings from this study will raise awareness about the existence of paternal depression in Pakistan and will assist researchers and policymakers to consider this issue while planning and implementing strategies to improve the mental health indicators in Pakistan.

## 5 CONCLUSION

Paternal postnatal depression is a prevalent but underresearched issue among Pakistani men. This issue is of concern because it has the potential to affect new fathers, their partners, and the newborn. There is an imminent need to incorporate fathers in the existing and future perinatal mental health programs, to raise awareness about paternal perinatal depression in Pakistan, to screen expectant and new fathers for depressive symptoms, and to support them during their transitioning to parenthood.

## 6 RELEVANCE FOR PRACTICE

Considering the health and socioeconomic costs of postnatal depression and its long‐term consequences on child development and well‐being, early recognition and management of risk factors for paternal postnatal depression are imperative. Because maternal depression was found to be a risk factor for paternal depression in this study, we recommend healthcare providers screen women during the antenatal period for possible depression, as maternal antenatal depression is one of the strongest predictors of maternal postnatal depression, which in turn may contribute to paternal depression and vice versa. Moreover, the positive association between paternal sleep disturbances and paternal postnatal depression highlights the need for healthcare providers to include men while planning educational and counseling programs to improve perinatal mental health in Pakistan. For this purpose, healthcare providers should encourage men to accompany their spouses for the antenatal visits, and use this opportunity to help new fathers in setting realistic expectations for their life during the postnatal period and to address any concerns regarding their new role as a father.

## AUTHOR CONTRIBUTIONS

Study design: Maria Atif, Mark Halaki, Chin‐Moi Chow. Data collection: Maria Atif. Data analysis: Maria Atif, Mark Halaki, Chin‐ Moi Chow, and Camille Raynes‐Greenow. Manuscript writing: Maria Atif, Mark Halaki, Chin‐ Moi Chow, and Camille Raynes‐Greenow.

## CONFLICT OF INTEREST

None declared.

## Data Availability

The data associated with this study is available from the corresponding author upon reasonable request.
